# Johnston’s Natural Philosophy

**Published:** 1846-07

**Authors:** 


### JOHNSTON'S NATURAL PHILOSOPHY.

The author of this manual is Professor of Natural Science in the Wesleyan University, and shows by the manner in which he has executed his task, that he understands how to exhibit the truths of science in a clear and striking light. It is an admirable text-book for students in high schools and academies. The publishers are Messrs. Thomas, Cowperthwait & Co. We owe a copy of it to the politeness of Messrs. Morton & Griswold.

